# Biological and therapeutic implications of RKIP in Gastrointestinal Stromal Tumor (GIST): an integrated transcriptomic and proteomic analysis

**DOI:** 10.1186/s12935-023-03102-6

**Published:** 2023-10-31

**Authors:** Nathália Cristina Campanella, Izabela Natalia Faria Gomes, Ana Laura Vieira Alves, Leticia Ferro Leal, Adriane Feijó Evangelista, Marcela Nunes Rosa, Matias Eliseo Melendez, Viviane Aline Oliveira Silva, Richard Lucas Konichi Dias, Lucas Faria Abrahão-Machado, Iara Santana, Olga Martinho, Denise Peixoto Guimarães, Vitor Marcel Faça, Rui Manuel Reis

**Affiliations:** 1 Molecular Oncology Research Center, Barretos Cancer Hospital, Rua Antenor Duarte Villela, 1331, CEP 14784 400, Barretos, S. Paulo, 14784-400 Brazil; 2 School of Health Sciences Dr. Paulo Prata (FACISB), Barretos, 14785-002 Brazil; 3 Molecular Carcinogenesis Program, National Cancer Institute, Rio de Janeiro, 20231-050 Brazil; 4 Department of Pathology, School of Medicine, Federal University of Bahia, Salvador, 40110-909 Brazil; 5 Gonçalo Moniz Institute, Oswaldo Cruz Foundation (IGM-FIOCRUZ/BA), Salvador, 40296-710 Brazil; 6 Department of Pathology, Barretos Cancer Hospital, Barretos, 14784-400 Brazil; 7 ICVS/3B’s - PT Government Associate Laboratory, Braga, 4806-909 Portugal; 8 Life and Health Sciences Research Institute (ICVS), School of Medicine, University of Minho, Braga, 4710-057 Portugal; 9 Department of Endoscopy, Barretos Cancer Hospital, Barretos, 14784-400 Brazil; 10 Department of Biochemistry and Immunology, Faculdade de Medicina de Ribeirão Preto da Universidade de São Paulo, Ribeirão Preto, 14049-900 Brazil

**Keywords:** RKIP expression, Gene knockout, GIST, COL3A1, Collagen, Cell invasion, Migration, Proteomics, Transcriptomics

## Abstract

**Background:**

Gastrointestinal stromal tumors (GIST) represent a significant clinical challenge due to their metastatic potential and limited treatment options. Raf kinase inhibitor protein (RKIP), a suppressor of the MAPK signaling pathway, is downregulated in various cancers and acts as a metastasis suppressor. Our previous studies demonstrated low RKIP expression in GIST and its association with poor outcomes. This study aimed to expand on the previous findings and investigate the biological and therapeutic implications of RKIP loss on GIST.

**Methods:**

To validate the RKIP prognostic significance, its expression was evaluated by immunohistochemistry in 142 bona fide GIST cases. The functional role of RKIP was evaluated in vitro, using the GIST-T1 cell line, which was knocked out for RKIP. The biological and therapeutic implications of RKIP were evaluated by invasion, migration, apoptosis, and 2D / 3D viability assays. Additionally, the transcriptome and proteome of RKIP knockout cells were determined by NanoString and mass spectrometry, respectively.

**Results:**

Immunohistochemical analysis revealed the absence of RKIP in 25.3% of GIST cases, correlating with a tendency toward poor prognosis. Functional assays demonstrated that RKIP knockout increased GIST cells’ invasion and migration potential by nearly 60%. Moreover, we found that RKIP knockout cells exhibited reduced responsiveness to Imatinib treatment and higher cellular viability in 2D and 3D in vitro models, as assessed by apoptosis-related protein expression. Through comprehensive genetic and proteomic profiling of RKIP knockout cells, we identified several putative RKIP-regulated proteins in GIST, such as COL3A1.

**Conclusions:**

Using a multidimensional integrative analysis, we identified, for the first time in GIST, molecules and pathways modulated by RKIP that may potentially drive metastasis and, consequently, poor prognosis in this disease.

**Supplementary Information:**

The online version contains supplementary material available at 10.1186/s12935-023-03102-6.

## Background

Gastrointestinal stromal tumors (GIST) are atypical mesenchymal tumors that affect the gastrointestinal tract, predominantly the stomach (60%) and small intestine (25%), and less frequently in the mesentery, esophagus, colon, rectum, and omentum (15% collectively) [1, 2]. Previous studies estimate that 40–50% of GIST patients develop recurrent or metastatic disease [3, 4].

The development of GIST is attributed to mutations in major oncogenes such as *KIT* and *PDGFRA*, which activate downstream signaling pathways, including MAPK, PI3K/AKT, and STAT3 pathways [1]. The majority of GIST cases (80%) harbor mutations in exon 11 of the *KIT* gene, which have a robust response to treatment with the kinase inhibitor imatinib [5], followed by mutations in the *PDGFRA* gene (10%), often associated with low-risk GIST [6]. Additionally, in the *KIT/PDGFRA* wild-type subset of GIST, are described somatic *BRAF* and germinative SDHx mutations [7–9].

The Food and Drug Administration (FDA) approved tyrosine kinase inhibitors, including Imatinib (Glivec®, Novartis Pharmaceuticals), Sunitinib (Sutent®, Pfizer), and Regorafenib (Stivarga®, Bayer), as first-line treatment for GIST patients, and as a second and third-line treatment for those with resistant GIST [4, 10–12]. Nevertheless, several patients still develop disease progression or primary/secondary resistance, associated with poor prognosis [13]. The prognosis of GIST is currently evaluated based on three pathological features: tumor size, site of origin, and mitotic rate [14, 15]. Furthermore, although the primary mutations in the initial course of GIST are in *KIT/PDGFRA* genes, new molecular changes during tumor progression determine the different clinical presentation and outcomes of patients [16].

The Raf kinase inhibitor protein (RKIP), also known as PEBP1 (phosphatidylethanolamine binding protein 1), is expressed in almost all normal human tissues and acts as an endogenous inhibitor of the MAPK signaling pathway [17]. RKIP binds to subdomains I and II of the RAF-1 kinase domains, blocking the phosphorylation of residues Ser338 by PAK kinases, and Tyr340/341 phosphorylation by Src family kinases, which are required for activation of RAF-1 [18]. RKIP can also bind to MEK and ERK, preventing their phosphorylation and activation by RAF-1 and diminishing downstream ERK kinase signaling [17, 19]. It is known that RKIP can also suppress the activation of the nuclear factor Kappa B (NFkB) cell survival pathway by blocking the IkB inactivation, an inhibitor of NFkB [20]. In addition, RKIP regulation has been implicated in G-protein coupled receptors (GPCRs) and GSK3 signaling pathways [17, 21, 22].

In cancer, RKIP expression is reported to be low, and several studies showed that it could behave as a negative prognostic marker in prostate cancer, breast, colorectal, gastric, pancreatic, gliomas, and hepatocellular carcinoma revealing this gene as a tumor suppressor [21, 23–30]. Additionally, it is documented that loss of RKIP expression is not due to the promoter methylation, and some in vitro and in vivo studies have shown its importance in the modulation of cellular growth [31, 32], motility [33, 34], epithelial to mesenchymal transition (EMT) [35], differentiation [36], invasion, and tumor metastasis [23, 37]. In previous work, our group reported low expression of RKIP in 9% of GIST, which was associated with poor disease-specific survival [38].

In the present study, we intend to extend the previous RKIP expression data by increasing the GIST patients analyzed. Moreover, to understand its biological and therapeutic role, we performed the RKIP knockout (KO) in a GIST cell line. Furthermore, several in vitro assays, and integrated transcriptomic and proteomic analyses, allowed the identification of novel putative genes and pathways regulated by RKIP in GIST.

## Methods

### Tissue samples

Formalin-fixed paraffin-embedded tissue (FFPE) samples from 142 primary GIST were acquired from the Pathology Department of Barretos Cancer Hospital, São Paulo, Brazil. The tumor samples were formerly classified according to Fletcher et al. criteria four risk assessment [39]. Routine immunohistochemistry (IHC) was used to evaluate the expression of the S100 protein and of CD117, CD34, and Desmin for histological and tumor identification. The presence of mutations in *KIT* (9, 11, 13, and 17 exons), *PDGFRA* (12, 14, and 18 exons), and *BRAF* genes were previously evaluated by Sanger sequencing [40–42]. No mutations were found in the *BRAF* gene. The present study was approved by the local ethics committee (approval number: 554/2011) of Barretos Cancer Hospital. Due to the study’s retrospective nature, patient consent was not necessary.

### Cell culture

GIST-T1 is an Imatinib-sensitive cell line, derived from GIST of the stomach of a Japanese woman and was established by Takahiro Taguchi (Kochi University, Kochi, Japan), and acquired in Cosmo Bio LTA (USA, Catalog No: PMC-GIST01C). The cells were cultured in DMEM (Dulbecco’s modified Eagle’s medium – ThermoFisher) (DMEM) supplemented with 10% fetal bovine serum (FBS) (Sigma-Aldrich, St. Louis, MO, USA) and 1% penicillin/streptomycin (P/S) (Life Technologies, Carlsbad, CA, USA) at 37 °C under a humidified atmosphere containing 5% CO_2_.

For RKIP knockout (KO) in the GIST-T1 cell line, it was used a Kit from Santa Cruz Biotechnology based on CRISP/Cas9 technology. The cells were transfected with a control plasmid (HDR Plasmid, Sc-401,270-HDR-2) containing a non-coding scrambled RNA sequence, to obtain Negative Control cells (NC), and both with control and Cas9 plasmid (CRISPR/Cas9 KO Plasmid, sc-401,270) to obtain RKIP KO cell line. The transfections were done using UltraCruz Transfection Reagent (Santa Cruz Biotechnology, Dallas, Texas, USA), and for a Stable transfection, the cells were selected with 2 µg/ml of puromycin. After two weeks of selection, red fluorescent protein (RFP)-positive cells were further enriched by flow cytometry cell sorting (FACSAria II, BD Biosciences, New Jersey, USA).

### Immunohistochemistry analysis (IHC) for RKIP

Immunohistochemical staining analysis was carried out on 4-µm thick sections through the streptavidin-peroxidase complex (Novolink Polymer Detection System, Leica Biosystems Newcastle Ltd., UK). The slides were deparaffinized and rehydrated for heat-induced epitope retrieval with citrate buffer (pH 6.0). Rabbit polyclonal antibody against RKIP (dilution 1:600) (Merck Millipore, Danvers, Massachusetts, USA, ref. 07–137) was used to examine RKIP expression. The immune reaction was visualized by 3,3′-diaminobenzidine as a chromogen, and all sections were counterstained with hematoxylin. The immunostaining was double-blind and evaluated by experienced pathologists (IS and LAM), according to the intensity of staining, as described previously [40]. Thus, the negative cases were those with absent (-) or weak (+) staining, and positive cases were those with moderate (++) or strong (+++) staining. The RKIP knockout and negative control GIST-T1 cells were formalin-fixed and paraffin-embedded into a cell block to be used as negative and positive controls, respectively.

### Wound healing migration assay

Edited GIST-T1 cells (1.5 × 10^6^ cells/well) were cultured in 6-well plates until cells reached 80–95% confluency. Wound healing assays were performed as previously described by our group [43, 44]. Microscopic photos of the wounds were taken at 0, 24, and 48 h of culture using the Axio Vert A1 FL (Carl Zeiss®, Oberkochen, Germany) microscopy, with the 10X objective. The results were expressed as the mean percentage of migration ± SD when compared to the time point 0 h (considered as 0% of migration). Statistical analysis was conducted using GraphPad PRISM version 9 (GraphPad Software, Inc.). The results are representative of three independent assays.

### Matrigel invasion assay

The invasion potential of the edited GIST-T1 cell was evaluated using the BD BioCoat Matrigel invasion chambers Kit (BD Biosciences, New Jersey, USA), following the manufacturer’s instructions and, as previously described [45]. The edited cell line (2 × 10^5^ cells/well) was seeded in serum-free DMEM inside the Matrigel-coated inserts (8 mm pore-size) in 24-well plates, while the lower chamber contained DMEM with 10% FBS. After 24 h, the cells that invaded the lower surface of the Matrigel-coated membrane were fixed with 70% methanol and stained with Hematoxylin/Eosin (HE) [44]. The cells were then photographed and counted through the Image J software. The results were expressed as the mean number of RKIP KO invaded cells ± SD in comparison to the negative control cells. Statistical analysis was performed using GraphPad PRISM version 9 (GraphPad Software, Inc.). The assays herein presented were done in triplicate and represented as the mean values obtained from three independent experiments.

### KIT inhibitors response: IC_50_, cell cycle, and 3D assay

GIST-T1 edited cells were treated with increasing doses of Imatinib and Regorafenib (Sigma-Aldrich) for 72 h, being then cellular viability determined by the MTS reagent (Cell Titer 96 Aqueous One Solution Cell Proliferation Assay, Promega, Madison, Wisconsin, EUA), for which absorbance values were measured at 490 nm using an automatic microplate reader Varioskan (Thermo Fisher Scientific, Waltham, Massachusetts, EUA). The absorbance values were calibrated to the DMSO vehicle alone (considered as 100% viability) and the half maximal inhibitory concentrations (IC_50_) were obtained by nonlinear regression analysis using GraphPad PRISM version 9 (GraphPad Software, Inc., La Jolla, CA, USA), as previously reported [41, 42].

To determine the impact of treatment in the cell cycle, the edited GIST-T1 cells (1 × 10^6^ cells/well) were seeded and serum-starved for 12 h, and after were exposed to IC_50_ and 1 µM values of Imatinib for 72 h in DMEM (0.5% FBS). The cell cycle distribution (G1, S, and G2/M) was determined using the flow cytometry BD FACSCanto II (BD Biosciences, New Jersey, USA) and its own software (BD FACSDiva), as previously described [46]. For apoptosis assessment upon treatment, the cells (1 × 10^6^ cells/well) were plated in a 6-well plate and allowed to adhere for at least 24 h and then serum-starved in DMEM (0.5% FBS). Subsequently, the cells were exposed to IC_50_ and 1 µM of Imatinib, in DMEM (0.5% FBS), for 24 h. The cells were subsequently lysed for western blot analysis.

Finally, for 3D aggregation and spheres formation, there were used NanoShuttle™-PL magnetic particles (Nano 3D Biosciences Inc., Houston, Texas, USA), which bind to the cell membrane and induce sphere formation through magnetization, and plates for suspension culture (Greiner Bio-One and Nano3D Biosciences technology). GIST spheres were treated with Imatinib at IC_50_ and 1 µM concentrations for 72 h. CellTiter-Glo Luminescent Assay (Promega, Madison, Wisconsin, EUA) was used to measure Cell Viability, by luminescence quantification in an automatic microplate reader (Varioskan, Thermo Fisher Scientific, Waltham, Massachusetts, EUA). The data were normalized to the vehicle (DMSO), which was considered as 100% of viability, and expressed as the relative percentage of cellular viability.

The assays were done in triplicate and represented as the mean values obtained from three independent experiments.

### Western blot

For RKIP and COL3A1 expression analysis, the genetically edited GIST-T1 cells (5 × 10^5^ cells/well) were seeded in 6-well plates and allowed to adhere for at least 24 h. Western blotting was performed using SDS-PAGE gel electrophoresis, as previously described [47]. For RKIP detection was used a polyclonal antibody (1:1000 dilution, Merck Millipore ref. 07-137) and for COL3A1 a monoclonal (1:1000 dilution, Abcam ref. ab184993), both incubated overnight at 4 °C. For apoptotic proteins assessment it was used PARP (1:1000), caspase-7 and 3 (1:500), and anti-BAX (1:500) antibodies (Cell Signaling Technology, Danvers, MA, USA). As loading controls, it was used β-actin (8H10D10, 1:2000, Cell Signaling Technology, Danvers, MA, USA, ref. #3700), α-tubulin (clone AA2, dilution 1:5000, Merck Millipore ref. 05-661), and GAPDH (1:1000 dilution, Cell Signaling Technology, Danvers, MA, USA, ref. #2118). The immune detection was carried out using enhanced chemiluminescence (ECL) Western Blotting Detection Reagent (GE Healthcare, Chicago, Illinois, USA), in the automatic ImageQuant mini LAS4000 system (GE Healthcare Chicago, Illinois, USA).

Quantification of Western Blot results was performed using band densitometry analysis with Image J software. Relative protein expression results are shown as the ratio between the target proteins and the respective loading controls. The results are shown as the mean value achieved after the quantification of at least two independent assays.

### Transcriptomic analysis by nanostring

The NanoString nCounter® PanCancer Pathways, a customized panel of 770 genes transcripts distributed in 13 biological pathways, was performed in biological triplicates of RKIP KO and negative control cells, using the NanoString *nCounter* Elements™, as previously described [48, 49]. A total of 100 ng RNA was isolated from cells using the RNeasy Mini kit (Qiagen, New York, NY, USA) following the manufacturer’s instructions. After RNA isolation, samples were washed and digested with DNAse, followed by additional washes and sample elution. RNA concentrations were assessed by Qubit Fluorometric Quantitation (Thermo Fisher Scientific, Waltham, Massachusetts, EUA).

The nCounter® Digital Analyzer captured reporter probe counts, and raw data were collected and preprocessed using the nSolver™ Analysis Software v3.0 (NanoString Technologies). The NanoStringNorm package (version November 18th, 2015) [50] was employed for data preprocessing and normalization, and statistical analyses were performed using T-test and heatmaps design in the R environment (R Foundation) with the multitest and Complex Heatmaps packages (https://bioconductor.org/packages/release/BiocViews.html). The level of significance for all analyses was set at 5%. Genes with fold change FC ≥ ± 2 and p < 0.05 were considered significant.

### Proteomic analysis by mass spectrometry (LC-MS/MS)

The protein cell extracts from control and KO cells were obtained as described [51]. The cell lysate was centrifuged at 20,000 x g for 30 min at 4 °C, after sonication cycles (Sonicador Unique, São Paulo, Brazil). The protein concentration was determined by the Bradford method (Bio-Rad, Hercules, CA). Aliquots containing 100 µg of protein were digested, as previously described [52].

To obtain total protein extracts, samples were diluted to a final concentration of 1 µg/µL, and 3 µL of each sample was injected into a two-dimensional chromatography system (Waters Co). The system consisted of an ACQUITY UPLC M-Class BEH C18, 130Å, 5 μm, 300 μm x 500 mm column, followed by an ACQUITY UPLC M-Class HSS T3 1.8 μm, 75 μm x 150 mm column (Waters Co). The samples were eluted with 50% acetonitrile/water containing 0.1% in the first dimension and analyzed with a gradient of 7–85% acetonitrile/water containing 0.1% formic acid for 54 min in the second dimension, totaling three acquisitions for each sample. The Synapt G2-Si (Waters Co, Milford, Massachusetts, USA) mass spectrometer was used for acquisition with positive mode nanoESI ionization, HDMSE acquisition, and spectra range from 50 to 2000 m/z using a ramp of energy fragmentation from 19 to 53 V. The acquired data was analyzed using the Progenesis QI for Proteomics software (Waters Co), in which proteins were identified and quantified. The revised human database of Uniprot (downloaded in 2017) was used to identify the proteins. Carbamidomethylcysteine was used as a fixed modification and the oxidation of methionine as a variable modification. Only peptides containing at least 2 fragments per peptide and 7 fragments per protein were considered to identify the proteins. For quantification, the high 3 peptides method as described by Silva et al. was employed [53]. Statistical analyses were carried out as described above for the transcriptomic data. These analyses were performed in partnership with the Department of Biochemistry and Immunology, Faculty of Medicine of Ribeirao Preto, University of Sao Paulo, Brazil.

### *In Silico* analysis

The functional gene-set enrichment analysis of the differentially expressed proteins was performed using the graphical gene-set enrichment tool of ShinyGO V0.741 software (http://bioinformatics.sdstate.edu/go74/). For enriched pathways identification the KEGG category was selected and an adjusted p-value cut-off (FDR) of 0.05 was chosen as the significance threshold. Functional protein association network analysis was done using STRING v11.5 (https://string-db.org/) to assess the interaction between the enriched proteins. The protein-protein interaction (PPI) enrichment p-value below 0.05 was used to determine a significant enrichment of network interaction.

The cBioPortal for Cancer Genomics (http://www.cbioportal.org), which is a repository of cancer genomics datasets, was used to analyze RKIP and the putative target molecules in 3 different tumor types: Colorectal Adenocarcinoma (TCGA, PanCancer Atlas − 594 samples), Esophageal Adenocarcinoma (TCGA, PanCancer Atlas − 182 samples), and Stomach Adenocarcinoma (TCGA, PanCancer Atlas − 440 samples) databases. According to the TCGA guidelines (http://cancergenome.nih.gov/publications/publicationguidelines), this dataset has no limitations or restrictions. For expression analysis, log-transformed mRNA expression z-scores compared to the expression distribution of all samples (RNA Seq V2 RSEM) and protein expression z-scores (RPPA) data were assessed. Significant alterations in mRNA expression and protein expression were determined by the z-score threshold of ± 2. Spearman expression correlations were determined directly in the cBioPortal platform considering all the samples and used to determine the different correlations between the interest genes. The Spearman correlation values were expressed as a heatmap.

### Statistical analysis

Associations between molecular and clinical data from patients were analyzed using the χ2 test or Fisher’s test. Cumulative survival probabilities were calculated using the Kaplan-Meier method, and statistical significance was determined by the log-rank test using SPSS 19.0 software (IBM SPSS, Armonk, NJ, USA). For in vitro assays, single comparisons between the different conditions studied were done using Student’s T test, done with Graph Pad PRISM 9. The level of significance in all the statistical analyses was set at p < 0.05.

## Results

### RKIP expression and clinical impact in GIST

**Fig. 1:**
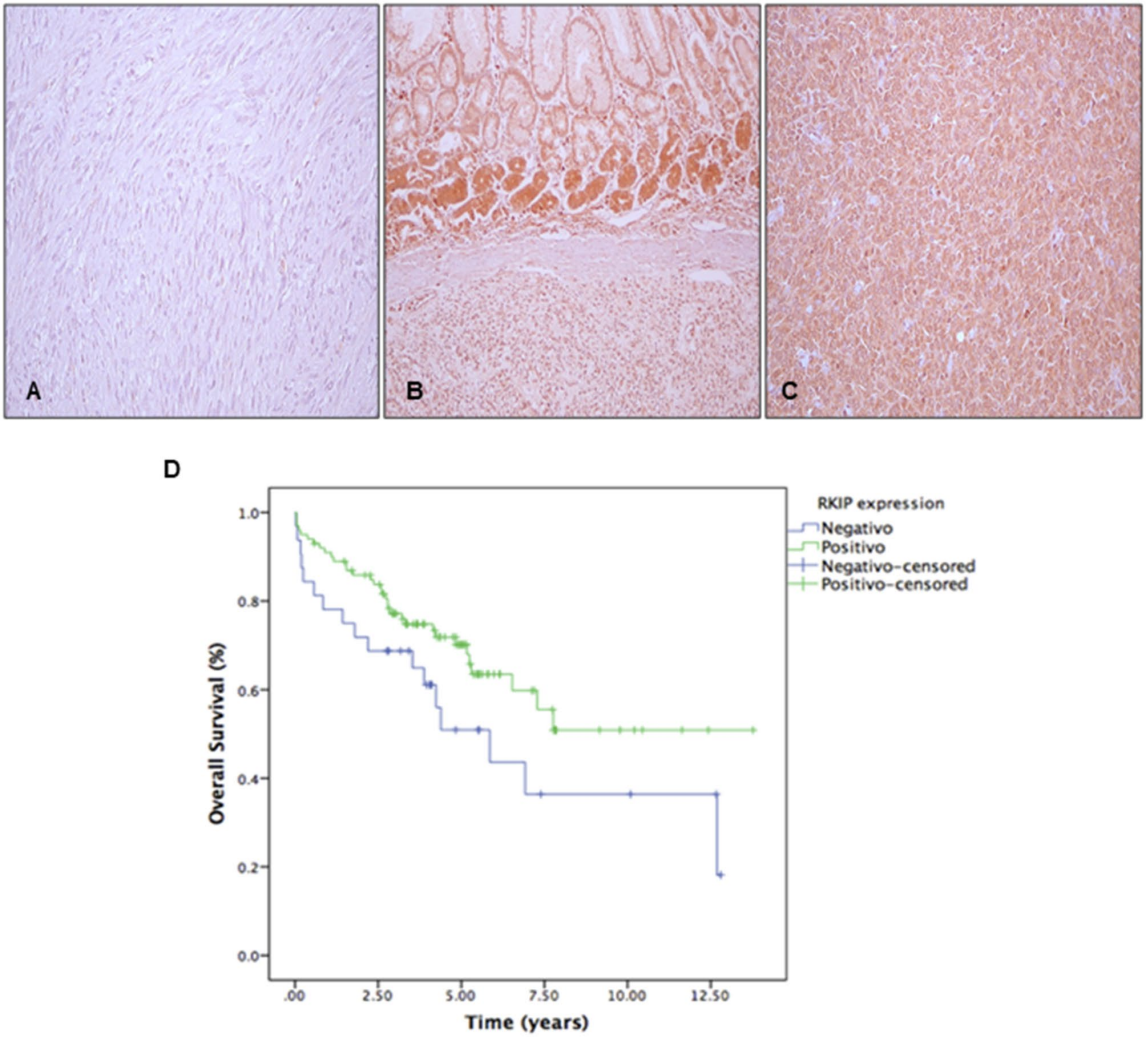
**Immunohistochemistry analysis of RKIP in GIST patient samples**. (**A**): negative expression (×200), (**B** and **C**): positive expression (×200). (**D**): Kaplan-Meier analysis for overall survival of GITS patients stratified by RKIP expression status (*p* = 0.077)

The immunohistochemistry analysis of RKIP in the 142 primary GISTs tissues showed low or absence of expression in 25.3% (36/142) of the cases, being classified as negative (Fig. 1A). The remaining 74.7% of the cases depicted moderate or high RKIP expression (Fig. 1B and C), being classified as positive.

The association between RKIP expression and clinical-pathological and molecular features is summarized in Suppl. Table S1. The Kaplan Meier analysis demonstrated that RKIP-negative patients tend to have a poor overall survival: in five years, the overall survival of RKIP-negative patients was only 50.9% compared to the RKIP-positive patients who showed a five-year survival rate of 70.2% (*p* = 0.077) (Fig. 1D). No other significant associations between RKIP and clinicopathological or molecular features were observed (Suppl. Table S1).

### RKIP knockout (KO) is associated with increased cellular invasion and migration in GIST-T1 cell line

**Fig. 2:**
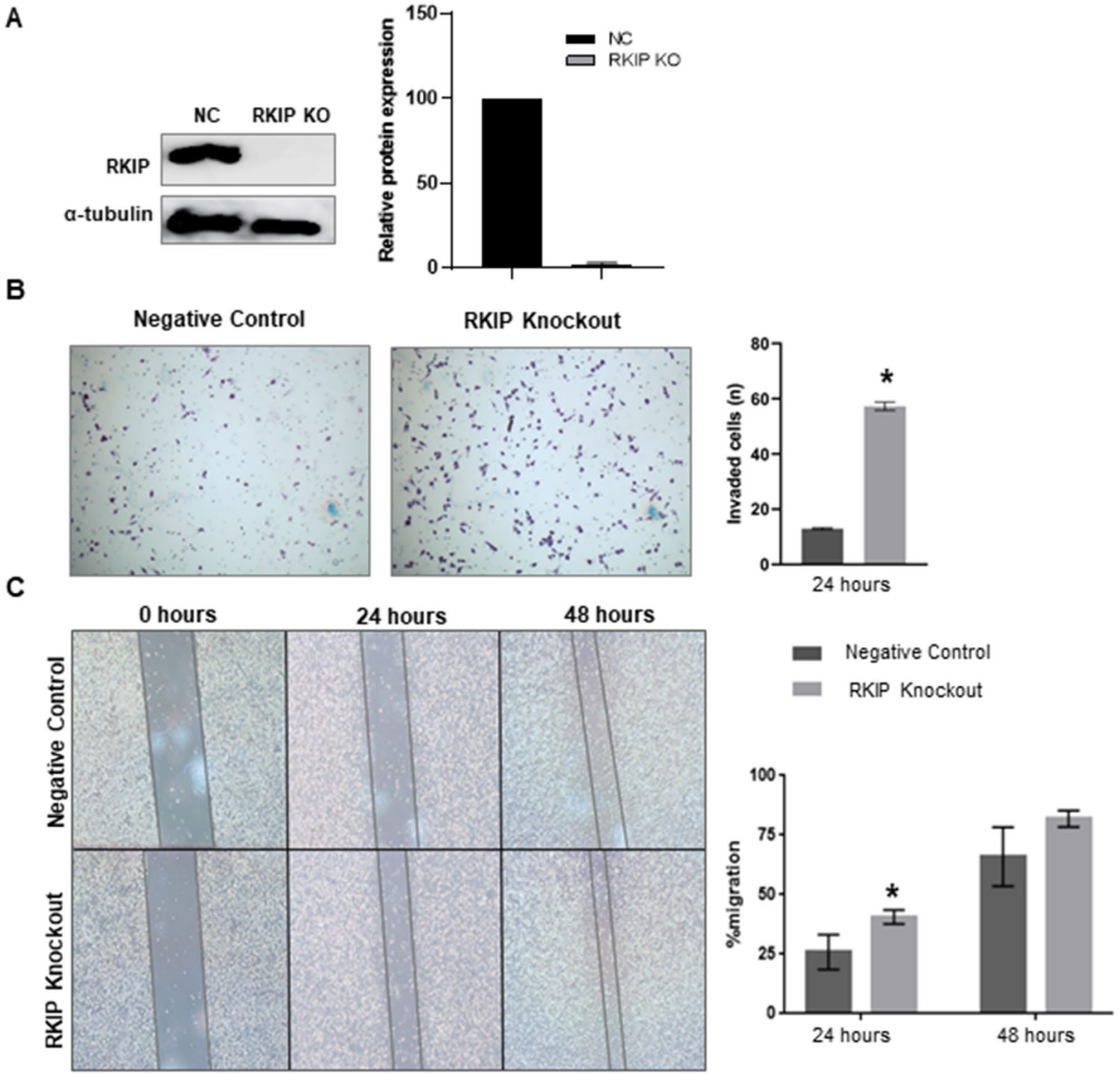
**Biological impact of RKIP KO in GIST-T1 cell line**. (**A**): Western Blot analysis of RKIP expression in GIST-T1 negative control (NC) and RKIP Knockout cells (KO). α-tubulin was used as the loading control. (**B**): Matrigel invasion assay in both GIST-T1 negative control and RKIP KO cells after 24 h at baseline. The results were expressed as the mean number of invading cells ± SD. (**C**): Cell migration of RKIP control and RKIP KO cells using Wound Healing Assay after 24 h, and 48 h at baseline. The results were expressed in relation to zero time point (considered as 0% of migration) and considering the mean percentage of migration ± SD. Both invasion and migration assays were done in triplicate and expressed as the mean of three independent experiments. Significance was considered at *p* < 0.05 (*)

To further explore the biological role of RKIP in GIST, we performed RKIP knockout (KO) in the GIST-T1 cell line using CRISPR/Cas9 technology. The efficiency of RKIP KO was confirmed by western blotting (Fig. 2A). The acquisition of a motile and invasive phenotype is an essential step in tumor progression and metastasis; therefore, we assessed the impact of RKIP KO on these biological features. We found that RKIP KO cells exhibited a significantly higher cell invasion, by matrix degradation within 24 h (*p* = 0.0006) (Fig. 2B). Additionally, we observed that the migration capacity of RKIP KO cells at the first 12 h was significantly higher than negative control cells (*p* = 0.031), while at 48 h, they migrate at a similar rate, but still RKIP KO cells tend to migrate faster (*p* = 0.099) (Fig. 2C).

### RKIP molecular signature in GISTs

**Fig. 3:**
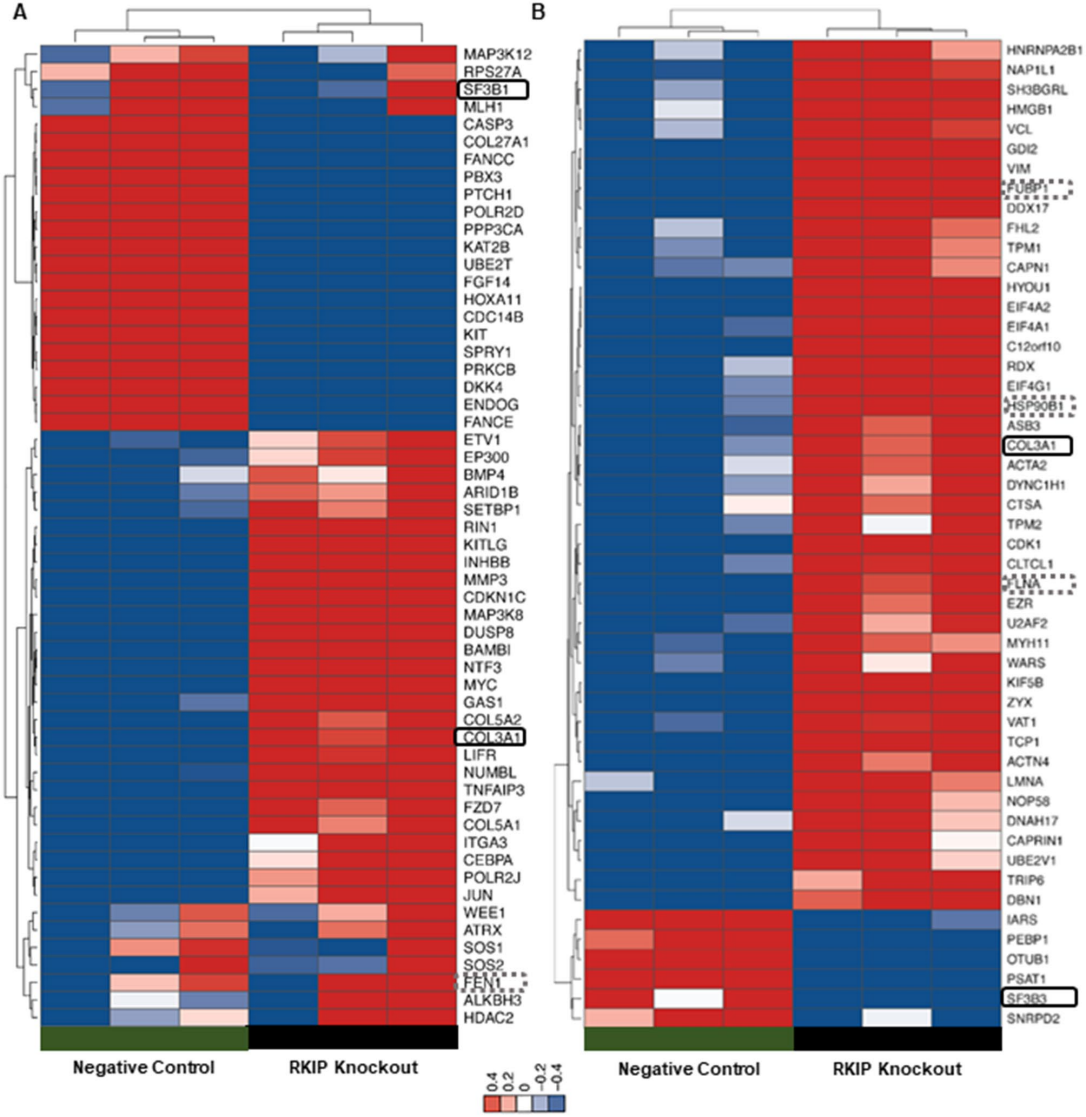
**Differentially expressed genes and proteins in the RKIP KO GIST-T1 cell line**. (**A**): Heatmaps showing at least two-fold upregulated (red) and downregulated (blue) genes and proteins (**B**), in the RKIP KO cells relative to negative control cells. Rectangles in bold are marking the common differentially expressed genes/proteins in both platforms, and the dot ones are showing other molecules that were differentially expressed in common, but with statistical differences in only one of the platforms

To further understand the network of molecules associated with RKIP downregulation in GIST, a transcriptomic and proteomic analysis (LC-MS/MS) was performed to pinpoint the genes/proteins differentially expressed in RKIP KO GIST-T1 cells (Fig. 3).

The transcriptomic profile was done using the nCounter® PanCancer Pathways panel (NanoString platform), which comprises 770 genes from 13 cancer-associated canonical pathways. The nCounter gene expression analysis identified 56 differentially expressed genes (Suppl. File S1), including 22 downregulated and 34 upregulated genes (Fig. 3A). Additionally, with a large-scale protein-based systematic analysis, by mass spectrometry, it was detected 506 proteins with a peptide count of at least > 3 (Suppl. File S1), with 42 of them being statistically significant differentially expressed: six down-expressed and 36 over-expressed (Fig. 3B and Suppl. File S1). The data was generated compared to the negative control, and an unsupervised clustering was performed to generate heatmaps of the groups, as described in the Methods section and depicted in Fig. 3.

The RKIP coding gene (*PEBP1*) is not present in the commercial nCounter® PanCancer Pathways (Fig. 3A), but, it was identified as significantly downregulated at the protein level, corroborating the KO experiments(Fig. 3B). We also found that vinculin (VCL) and vimentin (VIM) are among the most overexpressed proteins when RKIP is downregulated (Fig. 3B), which is in accordance with what is described for many other tumors [54]. Hence, the data suggest that RKIP KO in the GIST-T1 cell line mimics an RKIP loss phenotype.

**Fig. 4:**
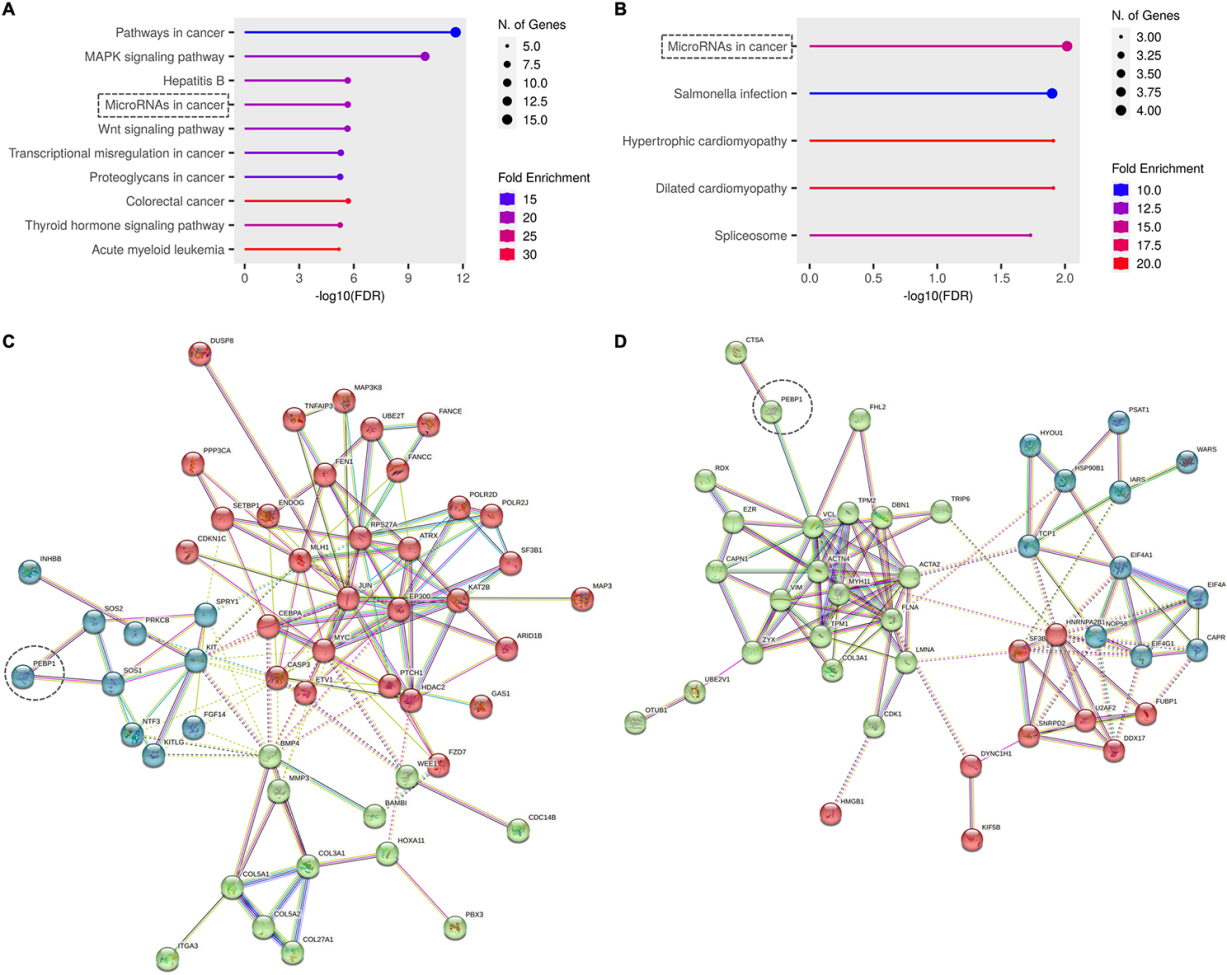
**Functional enrichment analysis of the genes and proteins differentially expressed in the RKIP KO GIST-T1 cell line**. Lollipop plots from the functional enrichment analysis done at ShinyGo 0.77 tool (http://bioinformatics.sdstate.edu/go/), showing up to 10 Top KEEG enriched pathways at mRNA (**A**) and protein level (**B**). Enriched pathways were sorted considering the -log10(FDR) values, the size of de circles is proportional to the gene numbers, and the color of the bars corresponds to the fold enrichment. (**C** and **D**): The functional protein association network was done in the STRING tool (https://string-db.org/), showing the 3 main significant interaction nodes within the network, at the mRNA and protein levels, respectively. Dot lines are showing the common pathways and signalizing the RKIP (PEBP1) position in the nodes

Moreover, we categorize the differentially expressed molecules by performing functional enrichment and protein association network analysis at the STRING (https://string-db.org/) platform (Fig. 4). We observed that the molecules found altered in RKIP KO cells belong to several enriched pathways (KEEG pathways), which are not fully concordant among genes and proteins: at the mRNA level there is an enrichment in cancer and MAPK associated pathways (Fig. 4A), while considering the MS results, the proteins found were associated with spliceosomes and microRNAs in cancer, being the last one also found among the enriched pathways at mRNA level (Fig. 4B). It is noteworthy that, both at the mRNA and protein level, there is a high level of interaction between the altered molecules, demonstrating functionality (Fig. 4C and D). Clustering the data by the three most functional interacting nodes, it is evident at the mRNA level that RKIP is not included in the main node (Fig. 4C - Red), which clusters genes associated with cell cycle, DNA repair, RNA biosynthesis, among others (Suppl. Figure S1). At the protein level, RKIP belongs to the main functional node (Fig. 4D - Green), associated with cytoskeleton organization, actin filaments and cell contraction, epithelial cell development, and differentiation (Suppl. Figure S1).

### COL3A1 as a novel RKIP-regulated molecule

The overlap analysis of transcriptome and proteome data revealed that 25 proteins were evaluated in common, from which six were found differentially expressed (Suppl. File S1). The proteins FLNA, HSP90B1, FUBP1, and FEN1 were found overexpressed in both analyses, however, only three got statistical significance at the protein level, while FEN1 got it at the mRNA level (Fig. 3 A). Curiously, the Splicing Factor 3B complex (SF3B) was found downregulated at both platforms, but the subunit 1 (SF3B1) at the mRNA level and the subunit 3 (SF3B3) at the protein level (Fig. 3 A). Remarkably, only one molecule, the protein COL3A1, was found to be significantly overexpressed in RKIP KO cells, when compared to the negative control, both at RNA and protein levels (Fig. 3 A).

**Fig. 5:**
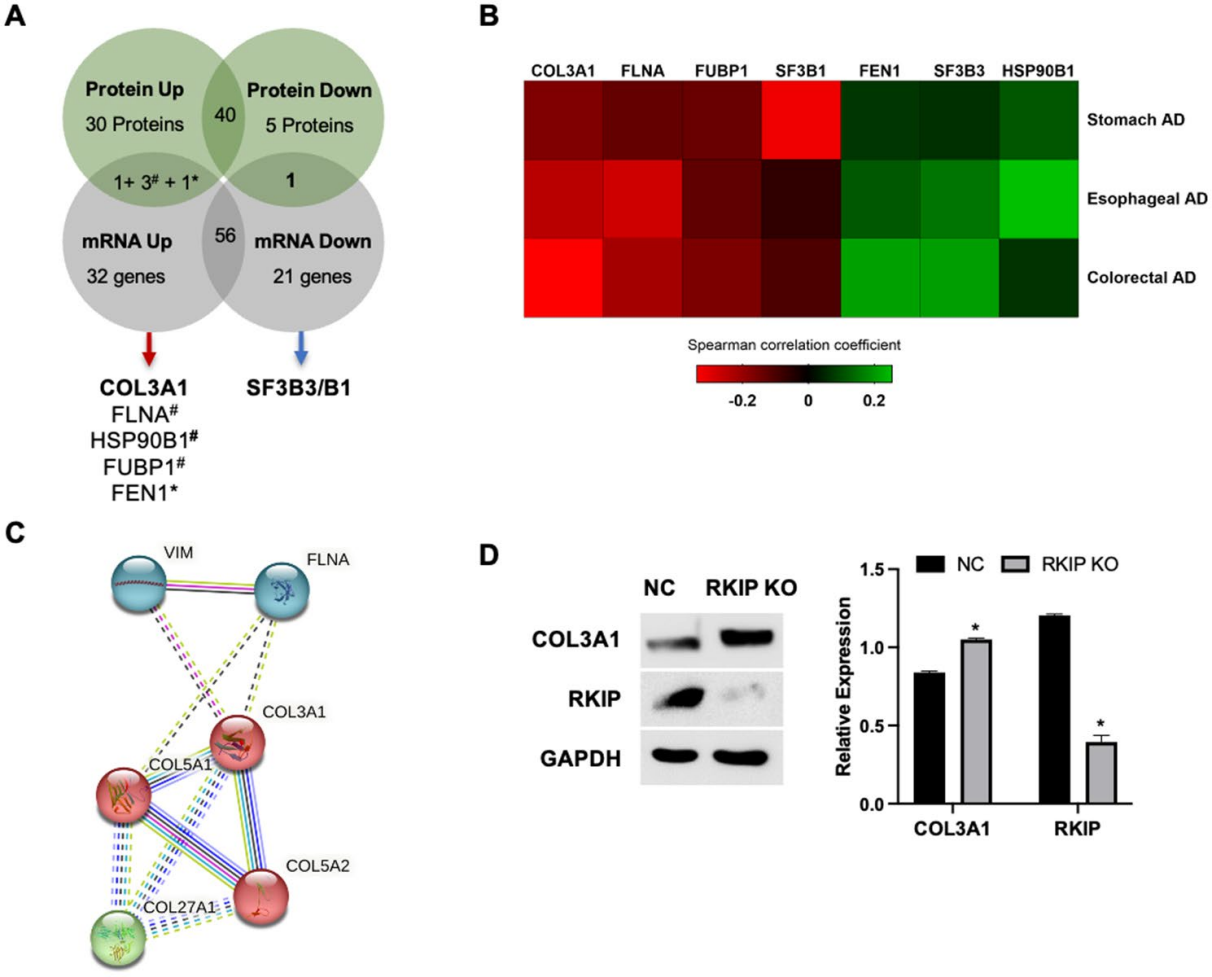
**COL3A1 is a novel putative target of RKIP in gastrointestinal tract tumors**. (**A**): Schematic representation of the differentially expressed genes found at the transcriptomic and proteomic analysis. (**B**): Using TCGA data, available at CBioportal (www.cbioportal.org), it was used the log RNA seq RPKM data from 3 TCGA PanCancer Atlas studies (Esophageal, colorectal, and stomach adenocarcinoma), to determine the correlation levels between RKIP (PEBP1) and the 6 commonly found altered molecules (A). The correlation plots and the Spearman correlation coefficients (Suppl. Table S2) are graphically represented as a heatmap, where PEBP1 is classified as positively (p > 0; dark to green) or negatively (p < 0; dark to red) correlated with the represented proteins. (**C**): Functional protein association network was done in STRING tool. (**D**): Western blot analysis of COL3A1 and RKIP expression in RKIP KO (Knockout) and NC (Negative Control) cells. GAPDH was used as a loading control, and COL3A1 expression was quantified as a ratio with GAPDH. The graph of relative protein quantification is expressed by the mean ratio with GAPDH, from two independent experiments. Significance was considered at *p* < 0.05 (*)

The TCGA data for GIST (Gastrointestinal Stromal Tumor (MSK, NPJ Precis Oncol 2023) has no information for gene expression, which hampered specific *in silico* validations. Thus, we wondered whether these proteins can also be correlated with RKIP in other tumor types, mainly the ones that arise in the same locations as GIST. To do so, we recurred to the TCGA PanCancer Atlas datasets for colorectal, esophageal, and stomach adenocarcinoma, and performed co-expression plots to determine the Spearman correlation levels between RKIP and the six genes cited above (Fig. 5B and Suppl. Table S2). Consistently with what we observed in the GIST-T1 cell line, COL3A1, FLNA, and FUBP1 genes showed to be inversely correlated, and SF3B3 positively correlated with RKIP expression in the datasets analyzed (Fig. 5B). However, across all the tumor types, the correlations were statistically significant only for *COL3A1* and *FLNA* genes (Suppl. Table S2).

Based on the enrichment analysis done above, it was interesting to note that, at the mRNA level, it were found expression alterations in other collagen coding genes, which are closely connected to COL3A1 (Fig. 4C), and that at the protein level, COL3A1 interacts directly with FLNA and VIM, being in the same node of interconnected proteins as PEBP1 (Fig. 4D). Altogether, COL3A1 protein showed to be the central mediator of a strong interconnection of proteins that are biologically associated with the extracellular matrix and collagen fibril organization (Fig. 5C). We confirmed by western blot analysis that RKIP KO cells have increased levels of COL3A1 when compared with control cells (Fig. 5D).

### Pharmacological impact of RKIP knockout in GIST

By transcriptomic analysis of GIST-T1 RKIP knockout cells, we observed that the KIT receptor was downregulated, while its ligand (KITLG) was overexpressed (Fig. 3A). Accordingly, we interrogated whether RKIP can be a modulator of GIST cells’ response to KIT-targeted therapies.

**Fig. 6:**
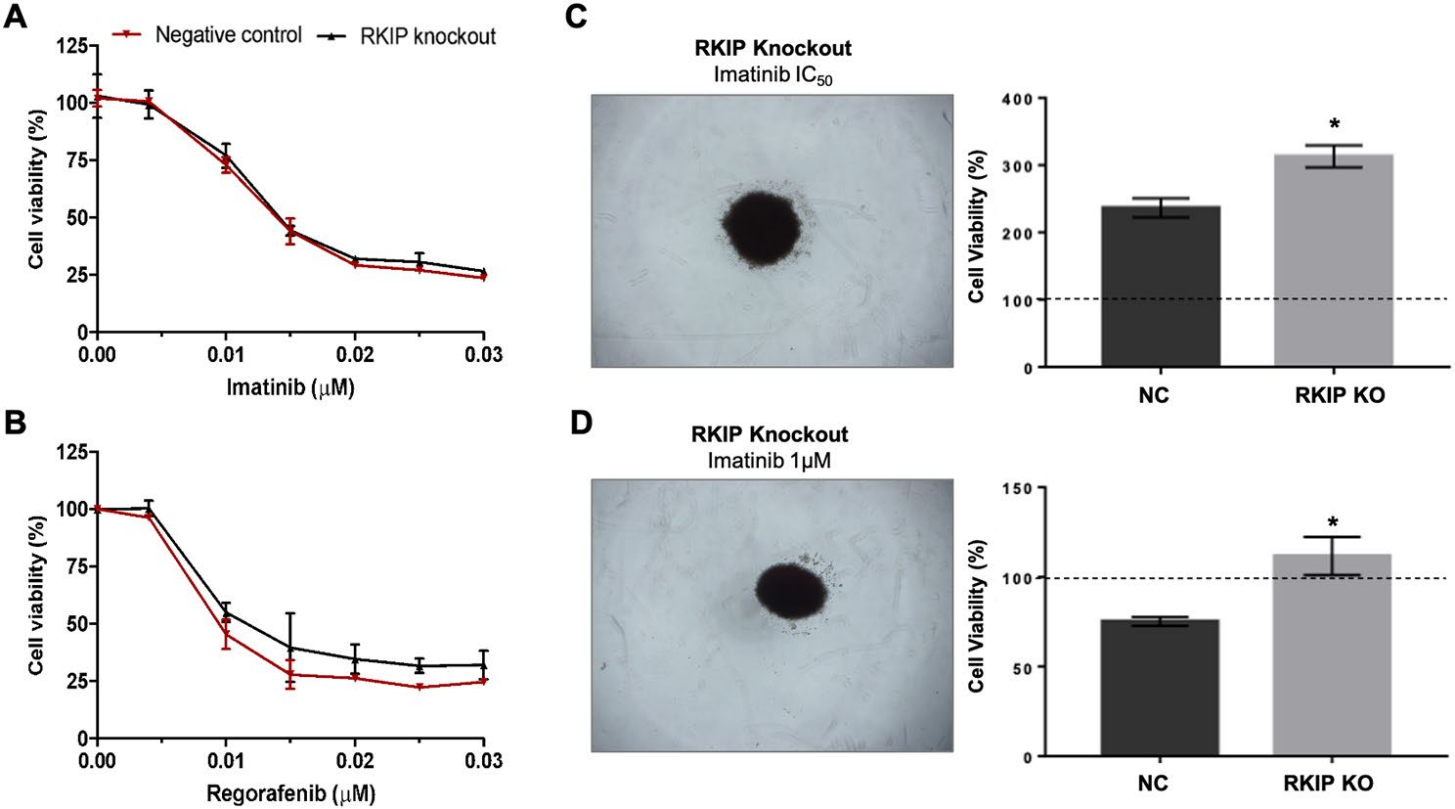
**Effect of RKIP on GIST-T1 cell line response to Imatinib and Regorafenib**. Representative dose-response curves of control and KO cells treated with Imatinib (**A)** and Regorafenib (**B**). The data are expressed as the percentage ± SD of viable cells, relative to the DMSO alone (considered 100% viability), and calculated as the mean of three independent assays, done in triplicate. (**C**): 3D in vitro proliferation model for control and RKIP KO cells, treated for 72 h with Imatinib at IC_50_ (0.015 µM) and at 1 µM concentration (**D**). The results show the mean percentage ± SD of viable cells in 3D spheres after imatinib treatment, relative to untreated cells (considered 100% viability - dot line). The experiment was done in triplicate and expressed as the mean of three independent assays. Significance was considered at p < 0.05 (*)

The effect of RKIP on cells’ response to Imatinib and Regorafenib was assessed by MTS assay, however, no significant differences were found (Fig. 6). Specifically, as can be verified in the dose-response curves, the mean IC_50_ value of Imatinib was 0.015 µM for both control and KO cells (Fig. 6A), and of Regorafenib was 0.011 µM and 0.014 µM for control and KO cells, respectively (*p* = 0.075) (Fig. 6B). Furthermore, it was implemented a 3D in vitro model to assess the potential of Imatinib in sphere formation disturbance. Following cells exposure to Imatinib, we observed that at the lowest concentration (IC_50_) there is no impact on spheres formation (Fig. 6C), with RKIP KO cells presenting a significant advantage when compared with the negative control (*p* = 0.0378). Treating the spheres with 1 µM of Imatinib (Fig. 6D ) resulted in cell toxicity on negative control cells (Cell Viability < 100%), but RKIP KO cells retained a significant potential for 3D sphere formation (*p* = 0.0419).

**Fig. 7:**
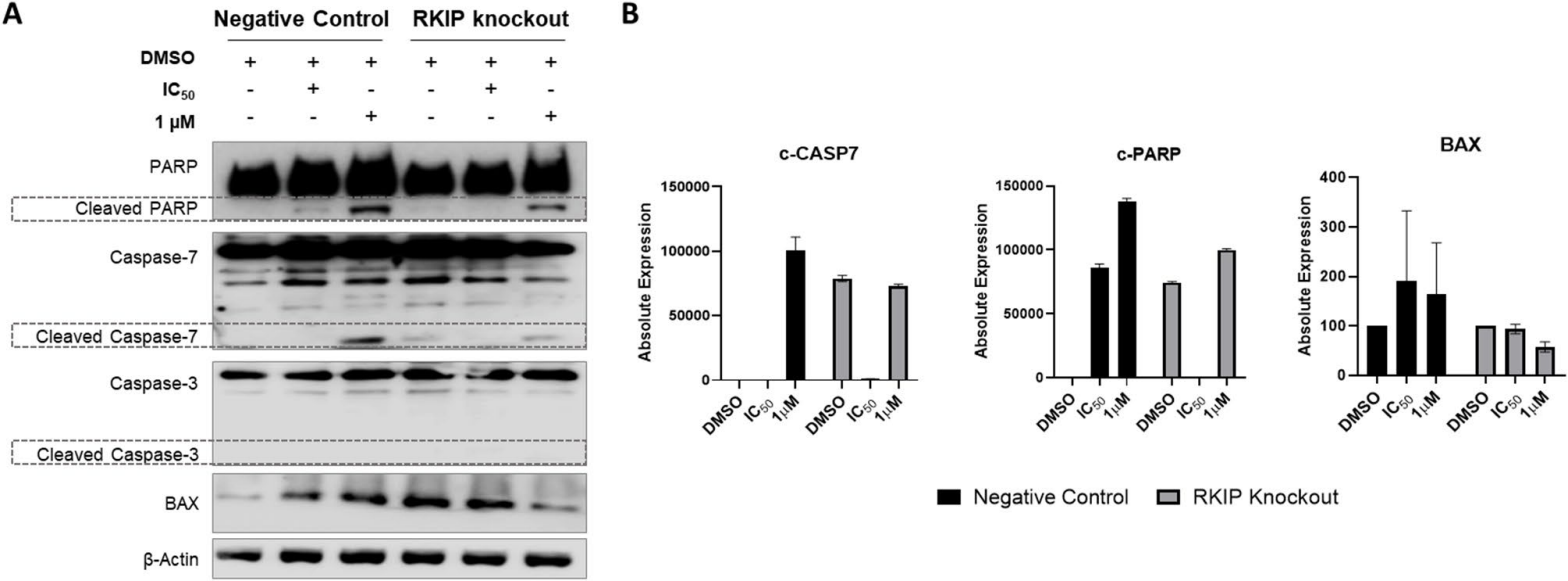
**Imatinib-induced apoptosis is abolished by the RKIP edition in GIST cells**. (**A**): GST-T1 cells, negative control, and RKIP KO cells were treated with indicated concentrations of imatinib for 24 h. Expression levels of apoptosis-associated proteins such as PARP, caspases 7 and 3, and BAX were assessed by performing a western blot. β-Actin was used as a loading control and the dotted line is indicating the proteins cleaved bands (**B**): Western blot quantification by bands densitometry, expressed as absolute quantification for cleaved PARP and Caspase-7 (CASP7), and as a ratio with β-Actin for BAX. The graphs are expressed as the mean of two independent experiments

Finally, we evaluated the apoptotic effect of RKIP in GIST cells exposed to Imatinib. The expression levels of PARP, caspase-7, caspase-3, and BAX were assessed by western blot before and after cells be treated with imatinib at IC_50_ and 1 µM concentrations (Fig. 7). Overall, it was found that Imatinib was effective in apoptosis induction at 1 µM, both in control and knockout cells, but with lower potency in RKIP KO cells (Fig. 7A). It is important to notice that was detected some residual expression of the cleaved forms of PARP and caspase-7, as well as high expression of BAX, in RKIP KO cells treated with DMSO (Fig. 7A), suggesting that some cells were becoming apoptotic probably due to its high proliferation rates in relation to the negative control cells. In fact, RKIP KO cells treated with low levels of Imatinib (IC_50_), present no cleavage of PARP nor CASP7, which means that grew slowly compared to the untreated KO cells, not suffering endogenous nor Imatinib-induced apoptosis (Fig. 7A and B). In contrast, PARP cleavage was detected in the negative control cells treated with the lower dose of Imatinib (Fig. 7B). Accordingly, at a higher dose of Imatinib (1 µM), the negative control cells presented high levels of PARP and CASP7 cleavage, as well as high levels of BAX induction, meaning sensitivity to KIT blockade (Fig. 7A and B). In RKIP KO cells, BAX expression decreased under imatinib-induced stress at high doses, suggesting lower responsiveness to KIT inhibition (Fig. 7A and B).

The effect of Imatinib on the cell cycle progression was also assessed, revealing that Imatinib exposure increases the number of cells in G0/G1 phase in both control and RKIP KO cells (Suppl. Figure S2).

Altogether, the results suggested that RKIP KO cells were less responsive to Imatinib-induced apoptosis, even presenting high levels of cell death at basal conditions.

## Discussion

The present study aimed to assess the prognostic value of RKIP protein expression in a large series of 142 Brazilian GIST and to investigate the biological and therapeutic impact of RKIP gene depletion in vitro using an integrated transcriptome and proteomic analysis.

GISTs are mesenchymal tumors that display constitutive activation of KIT or PDGFRA through gene mutations, which can predict the response to imatinib-based therapy [4, 11]. However, metastasis is common in GISTs, and about 40–50% of patients develop recurrent or metastatic disease, leading to a poor prognosis [4]. Despite this, the molecular markers for GIST prognosis remain poorly understood, emphasizing the need for identifying new markers such as RKIP [4, 11]. By performing immunohistochemistry analysis on 142 GISTs, we observed that RKIP expression was absent in approximately 25% of GIST tissues, and its loss was associated with a tendency toward poor prognosis. The loss of RKIP immunoexpression has been reported to range between 9 and 46% in previous studies [32–35]. Previous group work reported a loss of RKIP expression in approximately 9% of cases, which was associated with poor survival [38]. In contrast, in a study investigating 63 metastatic GISTs, RKIP downregulation was found in 14.5% of cases, and no influence on the patients’ survival was detected [55]. Schoppmann et al. evaluated 161 GISTs and showed that nearly 14% of tumors lacked RKIP expression [56]. A recent study evaluated 63 GISTs and reported that 46% of them exhibited negative RKIP expression. In this study, RKIP expression was significantly associated with higher tumor size, high risk according to Fletcher’s classification, mucosal invasion, and poor survival [57]. Although the frequency of RKIP loss in our study is consistent with the literature, the prognostic significance of RKIP is not entirely clear.

To understand the biological impact of RKIP loss, we used the CRISPR/Cas9 system to knock out RKIP in GIST-T1 cells. Our study demonstrated, for the first time, that the loss of RKIP increased three-fold the invasion potential and migration by nearly 60% in GIST cells. RKIP was shown to act as a tumor suppressor gene affecting negatively tumor cell survival, proliferation, and mainly metastasis [58]. Loss of RKIP expression has been an independent prognostic marker of poor outcome features of gastric, esophageal and colorectal cancer [59]. Also, RKIP loss is a predictive marker for the progression and metastasis of the liver and a survival indicator in lung cancer [60, 61]. Our findings suggest that RKIP does not modulate GIST in vitro viability response to Imatinib or Regorafenib. Nevertheless, we observed that in the absence of RKIP cells were less responsive to Imatinib-induced apoptosis, even presenting high levels of cell death at basal conditions. Therefore, further studies are needed to evalute the role of RKIP in GIST response to anti-KIT drugs.

To further elucidate which molecules may be drivers in GIST, we extensively analyzed the genetic and proteomic expression profile, comparing the RKIP knockout and control cell lines. Our analysis identified COL3A1 as being overexpressed at the mRNA and protein levels in RKIP KO cells. It is important to emphasize that although the proteomic data is on a global scale, the nanostring panel is focused on some specific pathways. Therefore, not all proteins found in the spectrometry were contemplated and could be related to the genes analyzed. In this study, we found other overlaps between genes differentially expressed at the mRNA and protein levels; however, the best targets chosen were based on criteria such as two-fold change and functional application within the model context. Thus, although COL3A1 was not the only target, it was considered the main one within our criteria of significant variation and functionality, corroborating its biological role within the study context.

The collagen Type III Alpha 1 Chain (*COL3A1*) gene, localized on the long arm of chromosome 2, encodes type III collagen [55]. Collagens are the main structural proteins of the ECM that interact with cells to regulate many functions, including differentiation, proliferation, and migration [62]. Although genetic modifications in tumor cells surely initiate and drive malignancy, cancer progresses within a dynamic remodeling of ECM [63]. Dysregulation of ECM structure, composition, abundance, and stiffness contributes to several pathological conditions, such as invasive cancer [62, 64]. It has been associated with increased mortality in breast, lung, and gastric cancer patients [65, 66].

Recently, COL3A1 was identified in association with the progression and prognosis of human bladder cancer [67]. Yuan et al. showed that patients with higher expression of COL3A1 had significantly shorter overall and disease-free survival [64]. In gliomas, Gao et al. determined that COL3A1 was increased in tumors, directly correlated with low grade, and conferred a survival advantage to patients [65]. In colorectal cancer (CRC), the upregulation of COL3A1 predicted poor overall and disease-free survival [68]. Moreover, a recent pan-cancer analysis, showed that COL3A1 is expressed in diverse tumor types and its expression is correlated not only with prognosis but with the immune microenvironment [69].

A recent miRNAs profiling of GIST identified let-7 as the most significant under-expressed miRNA in the worse prognostic subset of patients. The study pointed *COL3A1, COL5A2*, and *CASP3* as 3 out of its 4 targets in GIST [70], which are genes that were also found as differentially expressed in our RKIP silenced GIST cells. Interestingly, it was shown that RKIP can induce let-7, leading to the suppression of breast cancer metastasis [71, 72]. Further studies will be needed to address the cross-talk of RKIP, let-7 and COL3A1, and to deeply evaluate the role of COL3A1 as a novel RKIP-regulated gene.

## Conclusion

In summary, our study suggests that RKIP loss in GIST is associated with increased invasion and migration behavior. Additionally, using an integrative transcriptomic and proteomic analysis, we identified COL3A1 as a potential effector of RKIP in GIST.

### Electronic supplementary material

Supplementary Material 1

Supplementary Material 2

Below is the link to the electronic supplementary material.

Supplementary Material 3

Supplementary Material 4

Supplementary Material 5

## Data Availability

All data presented are contained within the manuscript.
