# Supplementary figures and images for: Biological and therapeutic implications of RKIP in Gastrointestinal Stromal Tumor (GIST): an integrated transcriptomic and proteomic analysis
Source: Cancer Cell Int. 2023 Oct 31;23:256. doi: 10.1186/s12935-023-03102-6 (PMC10619323; doi:10.1186/s12935-023-03102-6)

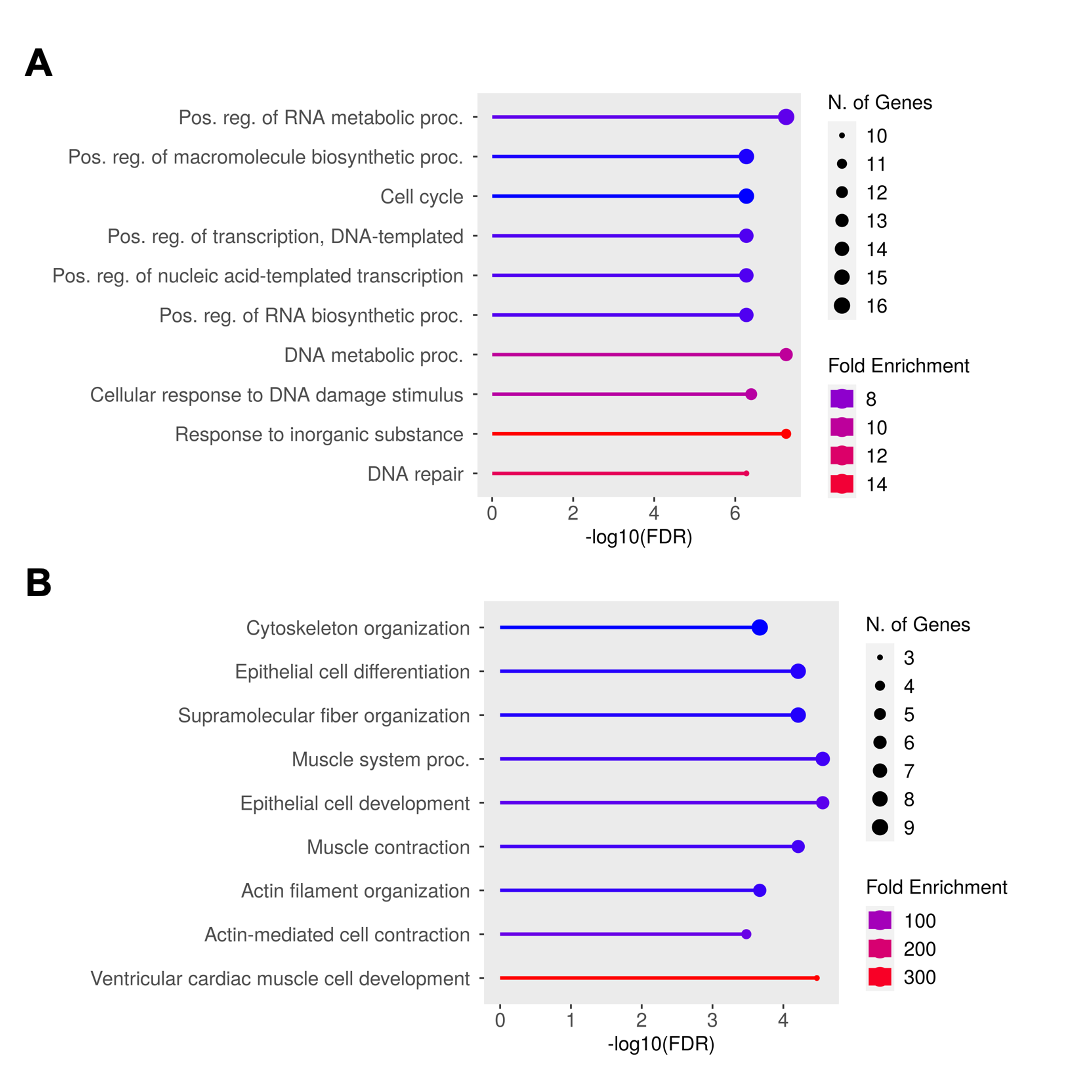

Supplement: Supplementary file 1 — Supplementary Material 1 [file 12935_2023_3102_MOESM1_ESM.png]

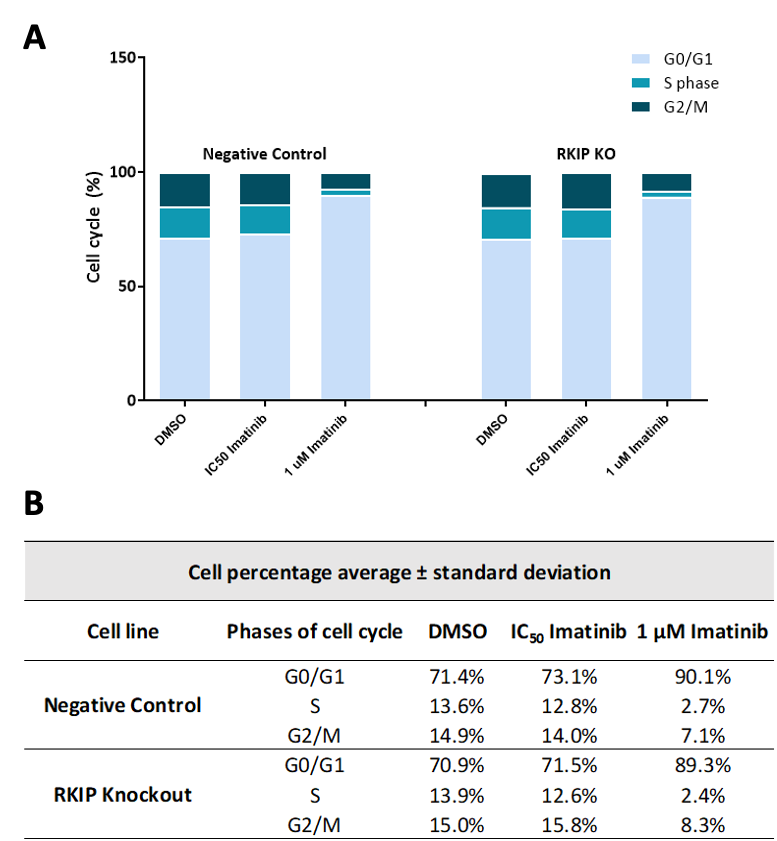

Supplement: Supplementary file 2 — Supplementary Material 2 [file 12935_2023_3102_MOESM2_ESM.png]
