# Supplementary material for: Biological and therapeutic implications of RKIP in Gastrointestinal Stromal Tumor (GIST): an integrated transcriptomic and proteomic analysis
Source: Cancer Cell Int. 2023 Oct 31;23:256. doi: 10.1186/s12935-023-03102-6 (PMC10619323; doi:10.1186/s12935-023-03102-6)
Supplement: Supplementary file 4 — Supplementary Material 4 [file 12935_2023_3102_MOESM4_ESM.docx]

**Table S1:** Association of RKIP expression with GIST clinicopathological and molecular features.

| **Parameter** | |  | | | |  | | | **N** | | | |  | | | | **RKIP expression** | | | | | | |  | | | | ***P* value** | | | |  | | | |
| --- | --- | --- | --- | --- | --- | --- | --- | --- | --- | --- | --- | --- | --- | --- | --- | --- | --- | --- | --- | --- | --- | --- | --- | --- | --- | --- | --- | --- | --- | --- | --- | --- | --- | --- | --- |
|  |  |  | | | |  | | |  |  |  |  |  | | | | Negative (%) | | | | Positive (%) | | |  | | | |  |  |  |  |  | | | |
| **Tumor localization** | | | |  | | | | | |  | | | |  | | | |  | | | |  | | |  | | | |  | | | |  |  |  |
|  | Small intestine |  | | |  | | | 44 | | | |  | | | | 15 (41.7) | | | | 29 (27.4) | | |  | | | 0.298 | | | |  | | | |  |  |
|  | Stomach |  | | |  | | | 68 | | | |  | | | | 13 (36.1) | | | | 55 (51.9) | | |  | | |  | | | |  | | | |  |  |
|  | Esophagus |  | | |  | | | 4 | | | |  | | | | 0 (0) | | | | 4 (3.8) | | |  | | |  | | | |  | | | |  |  |
|  | Colorectal |  | | |  | | | 13 | | | |  | | | | 4 (11.1) | | | | 9 (8.5) | | |  | | |  | | | |  | | | |  |  |
|  | Other |  | | |  | | | 13 | | | |  | | | | 4 (11.1) | | | | 9 (8.5) | | |  | | |  | | | |  | | | |  |  |
| **Risk classification** | | | | | | |  | | | |  | | | |  | | | |  | | | |  | | | |  | | | |  | | | |  |
|  | Very low |  | | | |  | | | 10 | | | |  | | | | 3 (11.1) | | | | 7 (8.8) | | |  | | | | 0.427 | | | |  | | | |
|  | Low |  | | | |  | | | 19 | | | |  | | | | 2 (7.4) | | | | 17 (21.3) | | |  | | | |  | | | |  | | | |
|  | Intermediate |  | | | |  | | | 26 | | | |  | | | | 8 (29.6) | | | | 18 (22.5) | | |  | | | |  | | | |  | | | |
|  | High |  | | | |  | | | 52 | | | |  | | | | 14 (51.9) | | | | 38 (47.5) | | |  | | | |  | | | |  | | | |
| **Tumor size (cm)** | | | | | | |  | | | |  | | | |  | | | |  | | | |  | | | |  | | | |  | | | |  |
|  | <2 cm |  | | | |  | | | 10 | | | |  | | | | 3 (9.7) | | | | 7 (7.6) | | |  | | | | 0.215 | | | |  | | | |
|  | 2 - 5 cm |  | | | |  | | | 36 | | | |  | | | | 5 (16.1) | | | | 31 (33.7) | | |  | | | |  | | | |  | | | |
|  | 5 – 10 cm |  | | | |  | | | 37 | | | |  | | | | 13 (41.9) | | | | 24 (26.1) | | |  | | | |  | | | |  | | | |
|  | >10 cm |  | | | |  | | | 40 | | | |  | | | | 10 (32.3) | | | | 30 (32.6) | | |  | | | |  | | | |  | | | |
| **Local recurrence** | | | | | | |  | | | |  | | | |  | | | |  | | | |  | | | |  | | | |  | | | |  |
|  | Absent |  | | | |  | | | 116 | | | |  | | | | 32 (91.4) | | | | 84 (80.8) | | |  | | | | 0.191 | | | |  | | | |
|  | Present |  | | | |  | | | 23 | | | |  | | | | 3 (8.6) | | | | 20 (19.2) | | |  | | | |  | | | |  | | | |
| **Metastasis** | | |  | | | |  | | | |  | | | |  | | | |  | | | |  | | | |  | | | |  | | | |  |
|  | Absent |  | | | |  | | | 75 | | | |  | | | | 21 (58.3) | | | | 54 (51.9) | | |  | | | | 0.564 | | | |  | | | |
|  | Present |  | | | |  | | | 65 | | | |  | | | | 15 (41.7) | | | | 50 (48.1) | | |  | | | |  | | | |  | | | |
| **Actual status** | | |  | | | |  | | | |  | | | |  | | | |  | | | |  | | | |  | | | |  | | | |  |
|  | Dead |  | | | |  | | | 50 | | | |  | | | | 17 (53.1) | | | | 33 (33) | | |  | | | | 0.059 | | | |  | | | |
|  | Alive |  | | | |  | | | 82 | | | |  | | | | 15 (46.9) | | | | 67 (67) | | |  | | | |  | | | |  | | | |
| **Mutational status** | | | | | | |  | | | |  | | | |  | | | |  | | | |  | | | |  | | | |  | | | |  |
|  | *KIT* |  | | | |  | | | 106 | | | |  | | | | 29 (90.6) | | | | 77 (81.1) | | |  | | | | 0.454 | | | |  | | | |
|  | *PDGFRA* |  | | | |  | | | 12 | | | |  | | | | 1 (3.1) | | | | 11 (11.6) | | |  | | | |  | | | |  | | | |
|  | Wild type |  | | | |  | | | 9 | | | |  | | | | 2 (6.3) | | | | 7 (7.4) | | |  | | | |  | | | |  | | | |
